# Antioxidant, Antidiabetic, and Anti-Obesity Properties of Apple Pulp Extracts (*Malus domestica Bork*): A Comparative Study of 15 Local and Commercial Cultivars from Spain

**DOI:** 10.3390/biology12070891

**Published:** 2023-06-21

**Authors:** Adrián Millán-Laleona, Francisco Javier Bielsa, Eduardo Aranda-Cañada, Carlota Gómez-Rincón, Pilar Errea, Víctor López

**Affiliations:** 1 Department of Pharmacy, Faculty of Health Sciences, Universidad San Jorge, 50830 Zaragoza, Spain; amillanl@usj.es (A.M.-L.);; 2 Unidad de Hortofruticultura, Centro de Investigación y Tecnología Agroalimentaria de Aragón (CITA), 50059 Zaragoza, Spain; fjbielsa@cita-aragon.es (F.J.B.); perrea@aragon.es (P.E.); 3 Instituto Agroalimentario de Aragón-IA2, CITA-Universidad de Zaragoza, 50013 Zaragoza, Spain; cgomez@usj.es

**Keywords:** polyphenols, antioxidant, antidiabetic, obesity, apple, extract, fruit

## Abstract

**Simple Summary:**

Apples are one of the most important fruits worldwide. In addition to their interesting nutritional value, they are a source of bioactive compounds with health-promoting effects. The composition may differ in different genotypes so it is important to investigate whether local varieties may differ in terms of total phenolic content, antioxidant, and bioactive properties. In this study Spanish local varieties showed better behavior in terms of phenolic content, antioxidant activity and bioactivity than other commercial samples, acting as functional foods with antiradical, antidiabetic and anti-obesity potential.

**Abstract:**

Apples (*Malus domestica* Borkh.) have a great agricultural and economic impact worldwide; they also present an interesting nutritional value, and their consumption has been associated with beneficial health effects. In this study, 15 apple varieties (three commercial, 12 autochthonous genotypes) were collected from mountainous areas in Spain and were evaluated for their phenolic content, antioxidant, anti-obesity and antidiabetic activities. Quercetin was tested as the reference substance in bioassays due to its role as one of the most common flavonoids in apples and other vegetables. Total Phenolic Content (TPC) of apple pulp extracts was quantified using the Folin-Ciocalteu method. The antioxidant activity was determined by using 1,1-diphenyl-2-picrylhydrazyl (DPPH) scavenging and xanthine/xanthine oxidase (X/XO) scavenging assays. Antidiabetic and anti-obesity potential were evaluated by inhibition of alpha-glucosidase (α-GLU), advance glycation end products (AGEs) formation and pancreatic lipase. The results showed in general higher phenol content in autochthonous varieties than in commercial apple pulp extracts. Phenolic-rich extracts showed better antioxidant profiles and significantly inhibited AGEs production and the α-glucosidase enzyme in a dose-dependent manner. None of them showed pancreatic lipase inhibitory effects but in general, the genotype known as “Amarilla de Octubre” was the best in terms of TPC and bioactive properties.

## 1. Introduction

Type 2 diabetes and obesity are the most prevailing chronic metabolic diseases worldwide and their incidence is increasing at an alarming high rate [1]. Both diseases are closely related to sedentarism, obesity and non-healthy dietary habits leading to a disorder known as metabolic syndrome; many studies support the relationship between their prevalence with poor vegetable and fruit consumption [2,3]. Additionally, fruits and vegetables are a great source of fiber, linked to lower incidence of cardiovascular disease and obesity, vitamins, minerals and phytochemicals such as polyphenols [4].

Polyphenols are non-nutritive bioactive compounds naturally present in fruits and vegetables, even in popular drinks such as wine, coffee, or tea. A great body of evidence suggests that polyphenols are related to a good health state as these phytochemicals possess antioxidant and anti-inflammatory activities also linked to anti-aging effects. Polyphenols can be divided in subcategories such as phenols, phenolic acids, flavonoids, anthocyanins, cumarins, ligans, stilbenes and tannins among others; they are characterized by the presence of phenol rings in their structure, which confers the ability to scavenge free radicals. Nowadays they are also an essential part of food supplements, cosmetic products and pharmaceutical formulations for the maintenance of healthy ageing [5]. Many polyphenols are used in cosmetic products and food supplements, claiming antioxidant effects, while other phenolic compounds such as salicylic acid may exert anti-inflammatory effects. Hydroxycinnamic acids, flavan-3-ols, flavonols or anthocyanins are some of the main phenolic groups in apples. They have an important role in the modulation of their color, quality indicators and maturity [6,7].

Flavonols are probably one of the most widespread flavonoids in plants and the most prominent in foods are quercetin and kaempferol [8]. Some of them have shown potential effects as inhibitors of some digestive enzymes (amylases, glucosidases, lipases) in regulation of serum sugar levels [9,10]. Furthermore, quercetin appears to modulate some of the harmful effects associated with obesogenic diets and it has been associated with adipocytes browning [11,12]. Synergisms between different polyphenols in vegetables attempt to show interesting action mechanisms in relation with inhibition of enzymes (amylases, glucosidases, lipases) related to digestion. It is import to underline the role of these molecules as desirable bioactive compounds due to their biological potential [13].

There is currently a decreasing trend in apple consumption (similar to other fresh fruits) in Spain, losing 30% of consumption per person per year in the last 30 years, and standing at one of the lowest consumptions in Europe, far from the recommendations of the OMS (400 g. of fruits and vegetables a day). However, products processed from fresh fruits and vegetables continue the growth observed in previous years, with an upward trend. Increasing fruit consumption has been recommended for the primary prevention of many chronic and metabolic diseases, including type 2 diabetes and obesity [14]. The exact mechanisms of action of polyphenols in the human body have not been proven yet but there are many studies and data that support their role against chronic diseases [15].

According to Food and Agriculture Organization (FAO) apples are one of the main crops in Europe, as it is the main temperate fruit tree species both in area and production at the European level [16]. Although they are rich sources of phytochemicals, such as polyphenols, apple characterization shows many physical and nutritional differences between different varieties [17,18]. For example, non-commercial varieties present higher polyphenol content or less allergenic genotypes than fruits developed after the green revolution, when genetic improvement of vegetables was focused on [19,20]. Some varieties have been demonstrated in vitro inhibitory effects against the enzymes α-glucosidase, lipase, monoamine oxidase A, tyrosinase and acetylcholinesterase [21]. Those biochemical traits, as well as other agronomical characteristics, could also be interesting for a better understanding of apple genomes, particularly to elucidate causal genes associated with human wellness [22]. In this project, twenty-one traits of interest, such as basic fruit quality, antioxidant parameters, individual sugars, and organic acids, were assessed by 23 simple sequence repeat molecular markers; these results showed that the polyphenol content in apples may suggest that breeders may be able to improve the nutritional value of apples through marker assisted breeding (MAB) or gene editing. However, much remains unknown about local apples as well as their quality indicators in comparison with the commercial varieties [23].

In mountain areas of the Pyrenees (Spain), generations of farmers performing selection processes in their orchards and home gardens have given rise to a wide diversity of quality fruit-plant material that constitutes an extraordinary genetic heritage of germplasm. Local apple accessions recovered in these areas showed an important range of genetic variability, suggesting interesting differences in the physical and chemical composition and nutritional properties. Those factors stablish the basis for further breeding and a major interest on fruits of proximity.

In this comparative study we selected 12 Spanish autochthonous and three commercial apple varieties from Navarra and Aragon for a better understanding of their polyphenol content and their biological properties in terms of antioxidative, antidiabetic and anti-obesity potential.

## 2. Materials and Methods

### 2.1. Materials, Reagents, and Plant Samples

Apples were collected from mountainous areas of the Pyrenees (Spain) and recovered in collections located in Garcipollera (Huesca, Aragon) (Pinova, Verde Doncella, Royal Gala, Pomera del Pais, P Pomes agrias, Doncella de Martin, Esperiega, Helada, Borau 01 and Amarilla de Octubre) and UPNA, Public University of Navarre (Arraiza, De Mine, Gordoncha, M. Tomate and Ziordia). Selected apple fruits were collected between August and October at the ripening period of each cultivar. The pulp tissues (500 g) were frozen immediately and lyophilized. The extraction of bioactive compounds was achieved by using methanol in a 33:1 (*v*/*w*) mixture of solvent:lyophilized ground tissue and ultrasonication for 20 min at room temperature. Extracts were stored at −80 °C after elimination of solvent in a rotatory evaporator. Quercetin (Fluorochem, Barcelona, Spain) was tested as the reference substance.

### 2.2. Total Phenolic Content (TPC) Using Folin-Ciocalteu Assay

For the quantification of phenolic compounds in the apple pulp extracts, 50 μL of Folin-Ciocalteu reagent (Sigma-Aldrich, Barcelona, Spain) was mixed with 10 μL of the sample, 100 μL of Na_2_CO_3_ 20% (*w*/*v*) and 840 μL of distilled water. Samples were incubated for 30 min at room temperature, and their absorbance at 700 nm was measured on a Synergy H1 reader (BioTek^®^ Instruments, Inc., Winooski, VT, USA). The results were expressed as the mean of milligrams of gallic acid per gram (GAE7 mg) of dry weight extract.

### 2.3. Radical Scavenging Activity by 2,2-Diphenyl-1-Picrylhydrazyl (DPPH) Assay

The reduction of the DPPH (Sigma-Aldrich, Barcelona, Spain) radical in the presence of the extract samples was carried out in 96 fluorescence microplates. DPPH solution (0.04 mg/mL) was prepared in HPLC quality methanol (Fisher Scientific, Barcelona, Spain) and protected from light before use. 150 µL of different extract samples concentrations were mixed with 150 µL of DPPH solution and released in a final volume of 300 µL in 96 fluorescence microplates. The neutralization of the radicals was measured at 515 nm after 30 min of dark incubation at room temperature [24]. Blank samples were considered, and percentages of inhibition were calculated according to the following formula (Equation (1)):

(1) $$Inhibition\left(\%\right)=\left[\frac{\left(Abs\;control-Abs\;sample\right)}{Abs\;control}\right]\times 100$$

### 2.4. Antioxidant Activity by Xanthine/Xanthine Oxidase (X/XO) Assay

The samples’ effects on the superoxide radicals (O_2_.) production of the X/XO system were measured by the NBT (nitro tetrazolium blue chloride) (Sigma-Aldrich, Barcelona, Spain) radical superoxide complex formation. The assay was developed using xanthine oxidase from bovine milk (Sigma-Aldrich, Barcelona, Spain). 16 mM Na_2_CO_3_ (Fisher Scientific, Barcelona, Spain), 22.8 µM NBT and 90 µM xanthine (Sigma-Aldrich, Barcelona, Spain) were dissolved in phosphate buffer NaH_2_PO4 (Fisher Scientific, Barcelona, Spain) 18 mM pH 7 to reach a volume of 240 µL on each well of a 96 microplate. Then, 30 µL of each sample, previously dissolved in the phosphate buffer, and 30 µL of xanthine oxidase (168 U L^−1^) were added to start the reaction. Assay mixtures were allowed to react during 2 min at 37 °C. After this incubation, plates were measured at 560 nm [25]. Blank samples were also considered, and percentages of inhibition were calculated according to Equation (1).

### 2.5. Alpha-Glucosidase (α-GLU) Inhibition Assay

The enzyme inhibition was evaluated using α-glucosidase from *Saccharomyces cerevisiae* (Sigma-Aldrich, Barcelona, Spain). The results were obtained using a 96-microplate reader. Each well contained 50 uL of sample, 100 uL of the enzyme (1 U mL^−1^) and 50 uL of 3 mM pNPG (4-nitrophenil alfa-d-glucopyranoside) (Sigma-Aldrich, Barcelona, Spain) as substrate. The absorbance was measured at 405 nm after 37 °C incubation and acarbose was use as reference inhibitor [26].

### 2.6. Advance Glycation End Products (AGEs) Production Assay

The inhibition of AGEs formation was measured fluorometrically in 96 black well-plates. Each well contained 50 µL of sample extracts, 100 µL of albumin solution (Santa Cruz, Barcelona, Spain) and 100 µL of fructose (Sigma-Aldrich, Barcelona, Spain). Plates were incubated for 24 h at 37 °C in the dark and then analyzed at an excitation wavelength of 355 nm and emission wavelength of 460 nm. To subtract intrinsic fluorescence resulting from incubation with BSA, a blank was made for each sample replacing the sugar with buffer. A blank control, with no AGEs formation, consisted of wells with only BSA and buffer [27,28].

### 2.7. Pancreatic Lipase Inhibition Assay

The capacity of extracts to inhibit pancreatic lipase (Sigma-Aldrich, Barcelona, Spain) was measured in 96 well plates. Pancreatic lipase solution (2.5 mg mL^−1^ enzyme in 0.1 M phosphate buffer, pH 7.0) was centrifugated at 2000× *g* for 7 min immediately before use. 40 µL of extract solution was mixed with 40 µL of enzyme solution and 20 µL of substrate solution 10 mM of p-4-nitrophenyl butyrate (NPB) (Sigma Aldrich, Barcelona, Spain). After incubation for 10 min at 37 °C absorbance was read at 405 nm. Orlistat (TCI, Barcelona, Spain) 1 mg/mL was used as positive control.

### 2.8. Statistical Analysis

Each experiment was performed at least three times on different days and results were expressed as the mean ± standard error (SE) of different assays. GraphPad Prism v.8 (GraphPad Software, San Diego, CA, USA) was used to perform data analyses, nonlinear regressions, correlations and statistics.

## 3. Results

### 3.1. Determination of Total Polyphenol Content (TPC)

**Figure 1:**
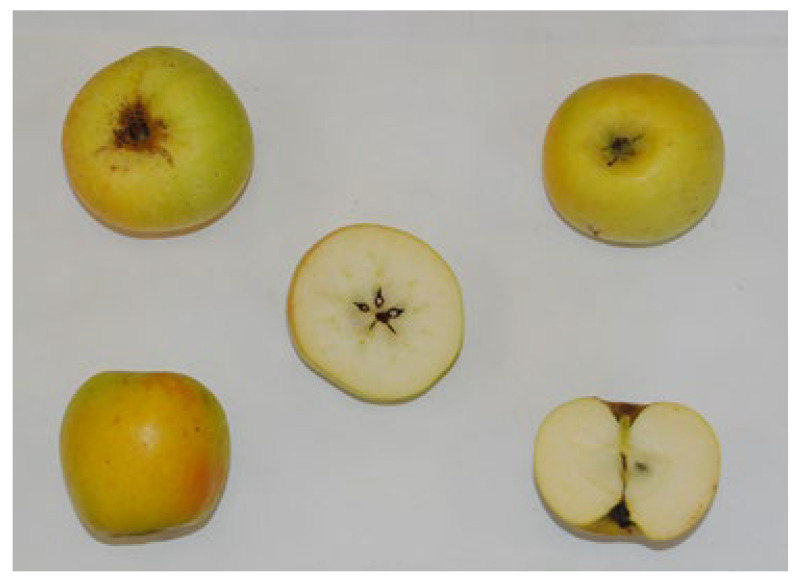
Samples of “Amarilla de Octubre” collected in the province of Huesca (Aragon, Spain).

**Figure 2:**
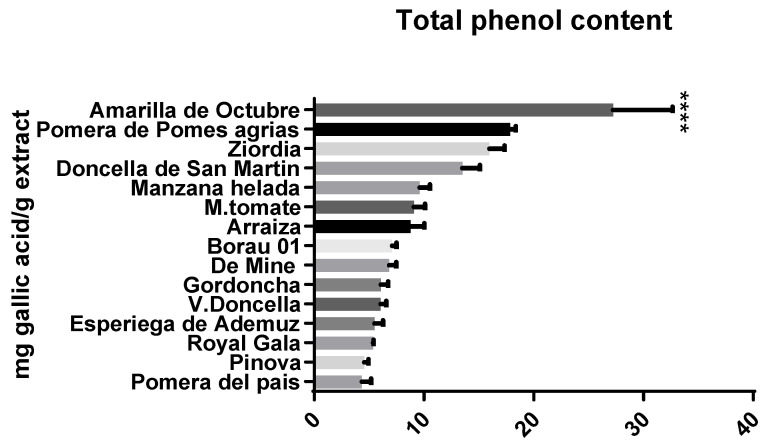
Total phenolic content evaluated by Folin-Ciocalteu method. Results are expressed as the mean (*n* = 4) of milligram of gallic acid per gram of apple pulp extract. Significant differences were marked between Ziordia, Pomera de Pomes agrias, Amarilla de Octubre, Doncella de San Martin and Pomera del pais and the commercial extract of V. Doncella. Note **** *p* < 0.001 versus V. Doncella. Data can be found as Supplementary Materials in Table S1.

Total phenolic content data were found by using the Folin-Ciocalteu method described in the materials and methods section. The apple extract with the highest polyphenol content was Amarilla de Octubre (Figure 1) showing significant differences against some of the other samples (Figure 2). Other extracts such as Pomera de Pomes agrias, Ziordia and Doncella de San Martin showed also high values with significant differences. Commercial genotypes such as Verde Doncella, Pinova and Royal Gala obtained low results (Available Data at Supplementary Materials).

### 3.2. Determination of Antioxidant Capacity

**Figure 3:**
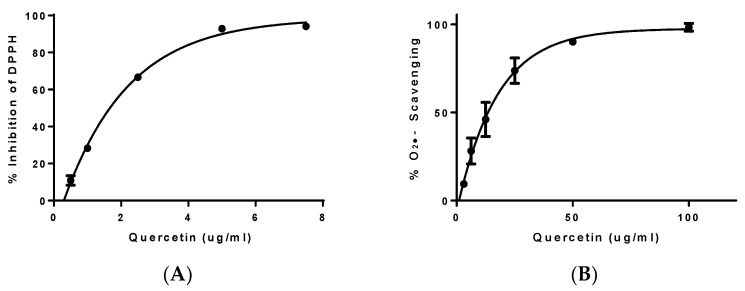
(**A**) DPPH inhibition percentage results expressed as the mean of different concentrations in micrograms per milliliter of quercetin. (**B**) X/XO scavenging assay expressed as the mean of different concentrations in micrograms per milliliter of quercetin. Note: Results are expressed as average ± SEM of at least three independent experiments.

Antioxidant properties were evaluated using DPPH and X/XO scavenging assays (Figure 3). Quercetin was used as the reference substance showing a good profile as radical scavenger (Figure 2); IC_50_ values for quercetin were 1.67 ± 0.06 (DPPH assay) μg/mL and 13.48 ± 0.95 μg/mL (X/XO assay).

DPPH and X/XO radical scavenging ability of extracts are shown in Table 1. Regarding the activity measured by both methods, Amarilla de Octubre and Pomera de Pomes agrias obtained similar IC_50_ results showing the greatest antioxidant activity. Other extracts such as Ziordia and Verde Doncella had also interesting results. None of them was able to achieve the IC_50_ results obtained with quercetin for the DPPH assay but Amarilla de Octubre was the most potent sample as a free radical scavenger. Quercetin was used as a reference compound because it is a common flavonoid in fruits and vegetables.

### 3.3. Determination of Antidiabetic Capacity

**Figure 4:**
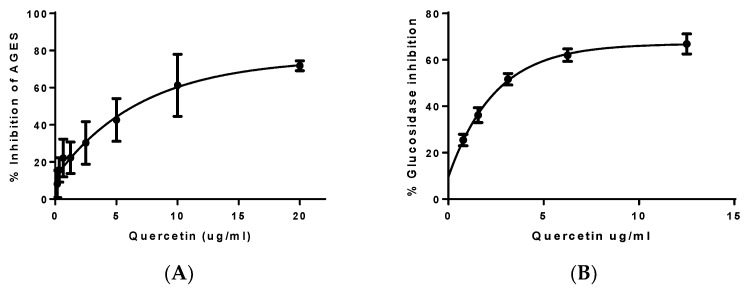
(**A**) AGEs inhibition percentage and (**B**) α-glucosidase inhibition percentage results expressed as the mean of different concentrations in micrograms per milliliter of quercetin. Note: Results are expressed as average ± SEM of at least three independent experiments.

Regarding the AGEs production assay, quercetin showed an IC_50_ of 6.42 ± 1.22 μg/mL (Figure 4A). Even though this concentration, quercetin was not able to achieve the total inhibition of the AGEs production. It also showed a strong inhibition of α-glucosidase with an IC_50_ of 2.98 ± 0.40 μg/mL (Figure 4B).

According to the α-glucosidase inhibition (Table 2), Amarilla de Octubre and Ziordia showed the lowest IC_50_ followed by Pomera de Pomes agrias, which continued to be one of the most interesting extracts. This tendency continued with the formation of AGEs (Table 2). In this experiment, Pomera de Pomes agrias and Amarilla de Octubre were again the most promising apples showing the lowest IC_50_. No activity was found in the Pomera del pais extract.

### 3.4. Determination of Anti-Obesity Capacity

**Figure 5:**
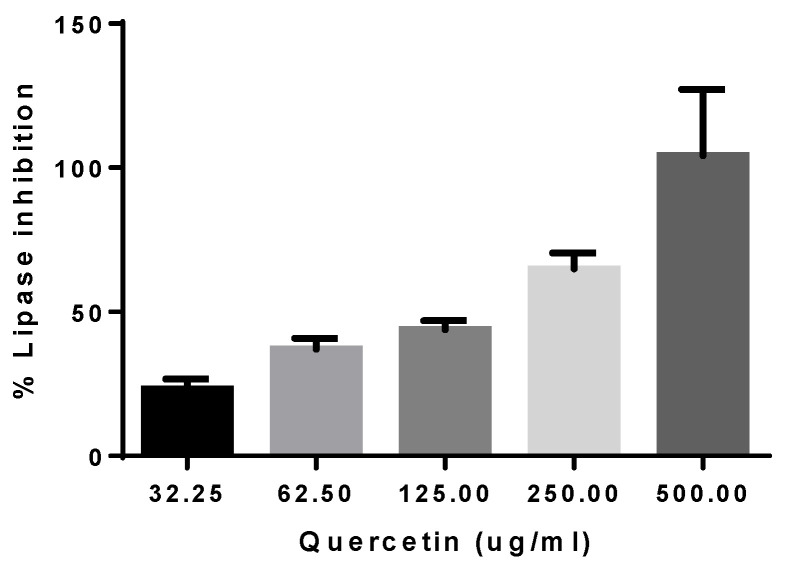
Lipase inhibition of quercetin expressed as the mean of different concentrations in micrograms per milliliter of quercetin. Note: Results are expressed as average ± SEM of at least three independent experiments.

In vitro inhibition of porcine pancreatic lipase was used to determine the anti-obesity potential of quercetin and the apple extracts. No inhibition was found for apple pulp extracts. However, quercetin showed an IC_50_ value of 156.63 ± 35.76 μg/mL (Figure 5).

### 3.5. Determination of Linear Correlation between Polyphenol Content and Bioactivity

**Figure 6:**
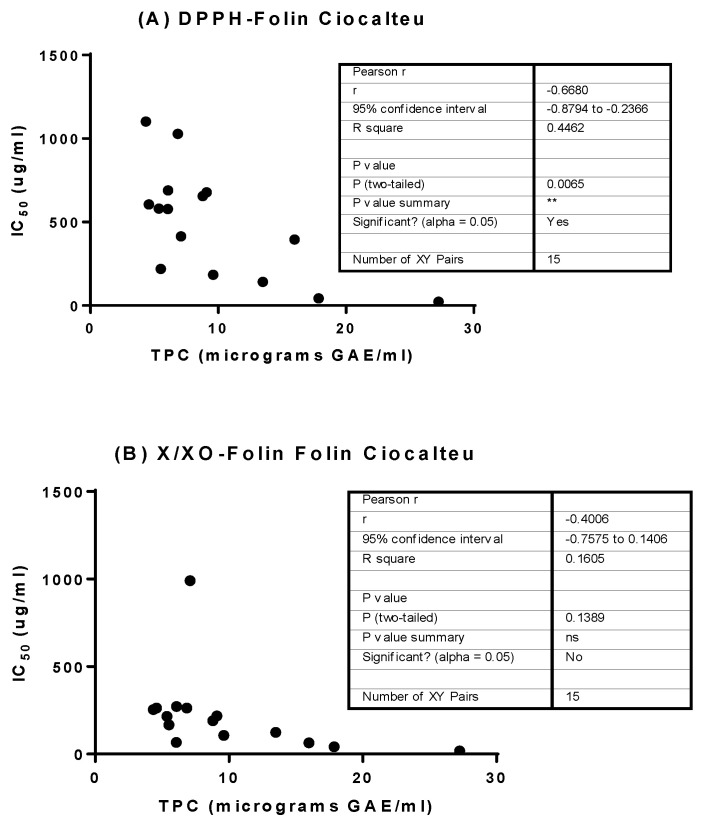

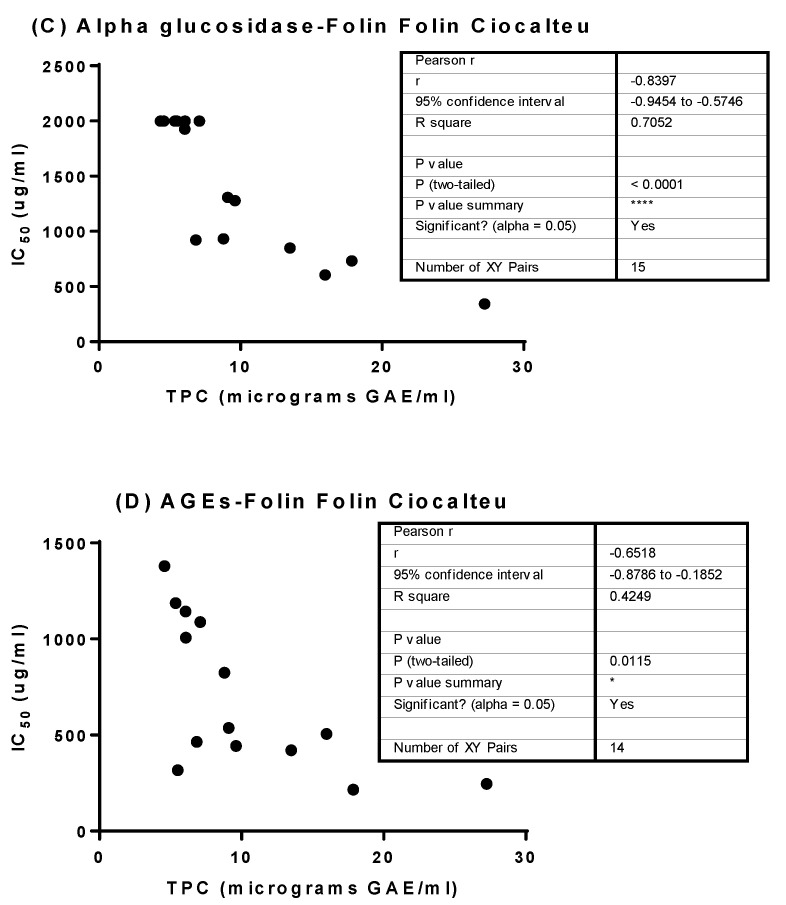
Correlation studies between polyphenol content and bioactivity. In (**A**,**C**,**D**) there are positive correlations between IC_50_ values and TPC, which seems to indicate that the antioxidant and antidiabetic activity may be mediated by polyphenols. * *p* < 0.05, ** *p* < 0.01, **** *p* < 0.001.

Linear correlation analysis between total polyphenol content (Folin-Ciocalteu method) and the IC_50_ of the bioassays was determined by Pearson correlation coefficient (Figure 6). Pearson r values confirm the relationship between DPPH, α-glucosidase and AGEs assays and polyphenol content suggesting that the antiradical, antidiabetic and anti-glycant activity may be mediated by polyphenols. However, no correlation was found for the X/XO assay. Correlation between TPC and lipase inhibitory activity could not be calculated as no lipase inhibition was found in our samples.

## 4. Discussion

Apples represents the fourth most important fruit eaten around the world and thousands of apple cultivars are grown to produce a variety of apples for the fresh market, and also for the food industry. Considering these facts, it is very important to identify which genotypes are more interesting for food consumption, not only considering taste and flavor but also the presence of bioactive compounds that could be involved in the prevention of metabolic and non-communicable diseases such as obesity and type 2 diabetes.

The Folin-Ciocalteu method was used in order to determine total phenolic content revealing a major presence of reducing phenolics in autochthonous apples compared to commercial varieties, as expected in previous studies with apples [20]. A lower presence of polyphenols in common varieties that are accessible to human consumption certifies the loss of an important source of those phytochemicals in the human diet [29]. It is important to consider that commercial fruits are usually selected for their appearance and the presence of oxidation in apple pulps is an important factor to avoid for marketing purposes. A lower production of colored polymers and different flavors caused for the contact between polyphenols and oxygen, in addition to enzymatic oxidation, could explain this tendency [30].

A quick and easy screening of antioxidant capacity of the samples was developed using the DPPH method. As complementary methodology and due to the importance of superoxide radicals in biological systems, X/XO complex scavenging assay was tested. Quercetin, a well-known flavonol with high antioxidant potential [31], showed a low IC_50_ as expected in both assays. Interestingly, antioxidative and anti-inflammatory effects have been reported also in vivo as well [32]. These effects of quercetin appear to be more highlighted when the basal levels of, respectively, the occurring oxidative stress and inflammation are high, such as in diabetes or obesity [33]. Regarding the extracts, our results showed that the samples from the region of Aragon named as Amarilla de Octubre and Pomera de Pomes agrias were the apples varieties with a major antioxidant potential; only Verde Doncella, was the commercial varieties with the lowest IC_50_ values in both DPPH and X/XO assays. Regarding this tendency, the regular consumption of autochthonous apples can be better source of antioxidants than commercial varieties.

The production of advanced glycation end products was strongly inhibited by quercetin. It is important to remark that AGEs are involved in the inflammatory and oxidative processes of certain tissues leading to ageing and insulin resistance. Previous studies related the consumption of dietary quercetin to a reduction in some AGEs precursors, such as methylglyoxal (MGO) and glyoxal (GO), in plasma and tissues [34]. Quercetin is supposed to trap those reactive dicarbonyl compounds regulating the detoxification systems and inhibiting AGEs production [35]. Apples showed higher IC_50_ than quercetin although they can achieve the total inhibition of AGEs production in comparison with quercetin. Regarding the pulp extracts, Amarilla de Octubre and Pomera de Pomes agrias had the better anti-glycative potential (IC_50_ 245.28 y 215.08 μg/mL, respectively). The total polyphenol content of the apple extracts seems to influence AGEs production. These results fit with many studies that found better antiglycan results in presence of two or more polyphenol substances [36].

Quercetin is an important inhibitor of α-glucosidase, one of the most important target enzymes in type 2 diabetes. Previous studies linked this effect for some flavonoids due to their interactions between their typical structures and some enzymes [10]. In general, apple extracts did not show strong enzyme inhibition potential except for Amarilla de Octubre (IC_50_ 341.89 μg/mL). This extract has a similar IC_50_ value than acarbose (373.59 μg/mL) [37], an important drug considered as the reference for glucosidase inhibitors in antidiabetic drugs. These results suggest the promising potential benefits of this genotype from Aragon. It is also interesting to remember that many bioactive and antidiabetic properties related to fruit consumption could be related to the inhibition of these enzymes. 

None of the apple pulp extracts showed an IC_50_ lower than 2000 μg/mL for the pancreatic lipase inhibition assay. We considered quercetin as a reference molecule, showing interesting inhibitory potential (Figure 5). Considering orlistat as reference drug in overweight and obese patients, other natural products with less unpleasant side effects should be explored. Recently, in silico tests linked flavonoids’ interactions with pancreatic lipase due to the relationship between the chemical/structural characteristics and the biological activity of the enzyme [38,39]. Our results put forward the idea that certain polyphenols such as quercetin or quercetin derivatives might be tested in in vivo experiments.

This manuscript suggests that local cultivars should be better explored in order to promote the consumption of local traditional varieties. This fact is in close relation with the Sustainable Development Goals (SDGs) established by United Nations for the 2030 Agenda for Sustainable Development, in which goal number 12 refers to “Responsible Consumption and Production”, focused on ensuring sustainable consumption and production patterns.

Apple pulp extracts from different genotypes and Spanish regions tested in this project showed quite different biological properties. Considering those assays, it seems that there is an interesting linkage between the phenolic-rich extracts and their antioxidant and antidiabetic properties. The autochthonous apple pulp extracts Amarilla de Octubre and Pomera de Pomes agrias showed the most interesting and promising results, suggesting the importance of protecting local autochthonous genotypes compared to commercial apples. Certain limitations are due to in vitro testing as bioactive properties should also be tested in other models using invertebrates or more complex organisms. Further studies are needed in order to elucidate which particular compounds are found in the different genotypes as well as to elucidate other beneficial properties related to healthy ageing. This study could be an interesting example of why the English proverb “*An apple a day keeps a doctor away*” may have a scientific sense.

## 5. Conclusions

This study highlights significant differences in the phenolic content and bioactive properties of 15 different apple pulp extracts, showing, in general, a more interesting profile in autochthonous varieties than the commercial apples. Pomera de Pomes agrias and Amarilla de Octubre extracts show the most interesting results in terms of polyphenol content and the lowest IC_50_ in the antioxidant assays. Thus, Amarilla de Octubre, Pomera de Pomes agrias and Ziordia had the most interesting antidiabetic potential as glucosidase and AGEs inhibitors. On the other hand, commercial varieties showed similar total polyphenol contents and bioactive activity. Regarding the commercial extracts, V. Doncella showed the highest total polyphenol content and the lowest IC_50_ in all the assays. However, no lipase inhibition was found in the apple extracts at physiological concentrations; quercetin has been revealed as an interesting flavonoid with antioxidant, antidiabetic and anti-obesogenic properties. Future studies will be performed in order to understand the compounds responsible for the activities as well as certain cellular and in vivo experiments might be developed. All these findings increase the interest in autochthonous fruits to promote the consumption of local apples due to their promising health benefits.

## Figures and Tables

**Table 1:** Results on antioxidant activity are expressed as average ± SEM of at least three independent experiments.

Sample	IC_50_ Values (ug/mL) in the Bioassays	
	DPPH	X/XO
Arraiza	655.38 ± 17.19	190.92 ± 20.18
De Mine	1027.94 ± 32.56	263.14 ± 12.72
Gordoncha	689.60 ± 21.39	272.48 ± 13.98
M. Tomate	678.33 ± 27.06	218.40 ± 11.99
Ziordia	395.91 ± 18.54	64.48 ± 3.24
Pomera de pomes agrias	43.36 ± 2.92	42.29 ± 3.70
Amarilla de Octubre	23.50 ± 1.34	18.40 ± 1.29
Doncella de San Martin	141.38 ± 11.40	124.71 ± 7.10
Pomera del pais	1101.67 ± 65.40	254.55 ± 17.92
Borau 01	414.03 ± 16.27	991.13 ± 145.80
Esperiega de Ademuz	218.76 ± 12.29	167.05 ± 32.92
Manzana helada	184.93 ± 10.49	107.20 ± 15.33
Royal Gala	580.73 ± 26.20	215.09 ± 28.13
Verde Doncella	578.04 ± 44.06	66.65 ± 6.71
Pinova	605.82 ± 26.06	263.25 ± 27.48
Quercetin	1.67 ± 0.06	13.48 ± 0.95

**Table 2:** Results of α-glucosidase inhibition are expressed as average  ±  SEM of at least three independent experiments.

Sample	IC_50_ Values (ug/mL) in the Bioassays	
	α-Glucosidase	AGEs
Arraiza	933.24 ± 298.00	823.79 ± 124.50
De Mine	922.20 ± 199.10	464.84 ± 81.85
Gordoncha	>2000	1006.47± 148.80
M. Tomate	1306.65 ± 211.00	536.78 ± 95.06
Ziordia	605.81 ± 161.40	506.10 ± 67.34
Pomera de pomes agrias	731.63 ± 42.49	215.08 ± 66.94
Amarilla de Octubre	341.87 ± 83.25	245.28 ± 36.73
Doncella de San Martin	848.45 ± 78.30	420.02 ± 61.64
Pomera del pais	>2000	-
Borau 01	>2000	1087.58 ± 117.10
Esperiega de Ademuz	>2000	316.71 ± 121.30
Manzana helada	1277.25 ± 135.40	443.09 ± 49.32
Royal Gala	>2000	1186.47 ± 126.50
Verde Doncella	1926.88 ± 275.70	1143.54 ± 140.90
Pinova	>2000	1379.56 ± 261.30
Quercetin	2.98 ± 0.40	6.42 ± 1.22

## Data Availability

The data presented in this study are available on request from the corresponding authors. The data are not publicly available due to privacy.

## Supplementary Materials

The following supporting information can be downloaded at: https://www.mdpi.com/article/10.3390/biology12070891/s1, Table S1: Folin-Ciocalteu data of the 15 apple pulp extracts expressed as the mean of milligrams of gallic acid per gram of dry weight extract of each repetition. Note: Outlier points are marked.

## References

[1] Dinda B., Dinda M., Roy A., Dinda S. (2019). Dietary plant flavonoids in prevention of obesity and diabetes. Adv. Protein Chem. Struct. Biol..

[2] Schwingshackl L., Hoffmann G., Lampousi A.-M., Knüppel S., Iqbal K., Schwedhelm C., Bechthold A., Schlesinger S., Boeing H. (2017). Food groups and risk of type 2 diabetes mellitus: A systematic review and meta-analysis of prospective studies. Eur. J. Epidemiol..

[3] Schwingshackl L., Hoffmann G., Kalle-Uhlmann T., Arregui M., Buijsse B., Boeing H. (2015). Fruit and Vegetable Consumption and Changes in Anthropometric Variables in Adult Populations: A Systematic Review and Meta-Analysis of Prospective Cohort Studies. PLoS ONE.

[4] Slavin J.L., Lloyd B. (2012). Health benefits of fruits and vegetables. Adv. Nutr. Int. Rev. J..

[5] Batiha G.E.-S., Beshbishy A.M., Ikram M., Mulla Z.S., El-Hack M.E.A., Taha A.E., Algammal A.M., Elewa Y.H.A. (2020). The Pharmacological Activity, Biochemical Properties, and Pharmacokinetics of the Major Natural Polyphenolic Flavonoid: Quercetin. Foods.

[6] Butkeviciute A., Viskelis J., Liaudanskas M., Viskelis P., Janulis V. (2022). Impact of Storage Controlled Atmosphere on the Apple Phenolic Acids, Flavonoids, and Anthocyanins and Antioxidant Activity In Vitro. Plants.

[7] Preti R., Tarola A.M. (2020). Study of polyphenols, antioxidant capacity and minerals for the valorisation of ancient apple cultivars from Northeast Italy. Eur. Food Res. Technol..

[8] Egbuna C., Awuchi C.G., Kushwaha G., Rudrapal M., Patrick-Iwuanyanwu K.C., Singh O., Odoh U.E., Khan J., Jeevanandam J., Kumarasamy S. (2021). Bioactive Compounds Effective Against Type 2 Diabetes Mellitus: A Systematic Review. Curr. Top. Med. Chem..

[9] Bule M., Abdurahman A., Nikfar S., Abdollahi M., Amini M. (2019). Antidiabetic effect of quercetin: A systematic review and meta-analysis of animal studies. Food Chem. Toxicol..

[10] Cao H., Chen X. (2012). Structures required of flavonoids for inhibiting digestive enzymes. Anti-Cancer Agents Med. Chem..

[11] Snyder S.M., Zhao B., Luo T., Kaiser C., Cavender G., Hamilton-Reeves J., Sullivan D.K., Shay N.F. (2016). Consumption of Quercetin and Quercetin-Containing Apple and Cherry Extracts Affects Blood Glucose Concentration, Hepatic Metabolism, and Gene Expression Patterns in Obese C57BL/6J High Fat–Fed Mice. J. Nutr..

[12] Gil Lee S., Parks J.S., Kang H.W. (2017). Quercetin, a functional compound of onion peel, remodels white adipocytes to brown-like adipocytes. J. Nutr. Biochem..

[13] Shahidi F., Dissanayaka C.S. (2023). Phenolic-protein interactions: Insight from in-silico analyses—A review. Food Prod. Process. Nutr..

[14] Muraki I., Imamura F., Manson J.E., Hu F.B., Willett W.C., van Dam R., Sun Q. (2013). Fruit consumption and risk of type 2 diabetes: Results from three prospective longitudinal cohort studies. BMJ.

[15] Williamson G. (2017). The role of polyphenols in modern nutrition. Nutr. Bull..

[16] Home|Food and Agriculture Organization of the United Nations. https://www.fao.org/home/en/.

[17] Kumar P., Sethi S., Sharma R.R., Singh S., Saha S., Sharma V.K., Verma M.K., Sharma S.K. (2018). Nutritional characterization of apple as a function of genotype. J. Food Sci. Technol..

[18] Podsędek A., Wilska-Jeszka J., Anders B., Markowski J. (2000). Compositional characterisation of some apple varieties. Eur. Food Res. Technol..

[19] Vegro M., Eccher G., Populin F., Sorgato C., Savazzini F., Pagliarani G., Tartarini S., Pasini G., Curioni A., Antico A. (2016). Old Apple (*Malus domestica* L. Borkh) Varieties with Hypoallergenic Properties: An Integrated Approach for Studying Apple Allergenicity. J. Agric. Food Chem..

[20] Jakobek L., García-Villalba R., Tomás-Barberán F.A. (2013). Polyphenolic characterisation of old local apple varieties from Southeastern European region. J. Food Compos. Anal..

[21] López V., Les F., Mevi S., Wandjou J.G.N., Cásedas G., Caprioli G., Maggi F. (2020). Phytochemicals and Enzyme Inhibitory Capacities of the Methanolic Extracts from the Italian Apple Cultivar Mela Rosa dei Monti Sibillini. Pharmaceuticals.

[22] Mignard P., Font i Forcada C., Giménez R., Moreno M.Á. (2023). Population Structure and Association Mapping for Agronomical and Biochemical Traits of a Large Spanish Apple Germplasm. Plants.

[23] Drogoudi P.D., Pantelidis G. (2011). Effects of position on canopy and harvest time on fruit physico-chemical and antioxidant properties in different apple cultivars. Sci. Hortic..

[24] López V., Akerreta S., Casanova E., García-Mina J.M., Cavero R.Y., Calvo M.I. (2007). In vitro antioxidant and anti-rhizopus activities of lamiaceae herbal extracts. Plant Foods Hum. Nutr..

[25] Rodríguez-Chávez J.L., Coballase-Urrutia E., Nieto-Camacho A., Delgado-Lamas G. (2015). Antioxidant Capacity of “Mexican Arnica” *Heterotheca inuloides* Cass Natural Products and Some Derivatives: Their Anti-Inflammatory Evaluation and Effect on *C. elegans* Life Span. Oxidative Med. Cell. Longev..

[26] Cásedas G., Les F., Gómez-Serranillos M.P., Smith C., López V. (2016). Bioactive and functional properties of sour cherry juice (*Prunus cerasus*). Food Funct..

[27] Spínola V., Castilho P.C. (2017). Evaluation of Asteraceae herbal extracts in the management of diabetes and obesity. Contribution of caffeoylquinic acids on the inhibition of digestive enzymes activity and formation of advanced glycation end-products (in vitro). Phytochemistry.

[28] Kim Y.S., Lee Y.M., Kim H., Kim J., Jang D.S., Kim J.H., Kim J.S. (2010). Anti-obesity effect of Morus bombycis root extract: Anti-lipase activity and lipolytic effect. J. Ethnopharmacol..

[29] Vrhovsek U., Rigo A., Tonon D., Mattivi F. (2004). Quantitation of Polyphenols in Different Apple Varieties. J. Agric. Food Chem..

[30] Zhou X., Iqbal A., Li J., Liu C., Murtaza A., Xu X., Pan S., Hu W. (2021). Changes in Browning Degree and Reducibility of Polyphenols during Autoxidation and Enzymatic Oxidation. Antioxidants.

[31] Salvamani S., Gunasekaran B., Shaharuddin N.A., Ahmad S.A., Shukor M.Y. (2014). Antiartherosclerotic Effects of Plant Flavonoids. BioMed Res. Int..

[32] Wilms L.C., Hollman P.C.H., Boots A.W., Kleinjans J.C. (2005). Protection by quercetin and quercetin-rich fruit juice against induction of oxidative DNA damage and formation of BPDE-DNA adducts in human lymphocytes. Mutat. Res. Toxicol. Environ. Mutagen..

[33] Boots A.W., Haenen G.R.M.M., Bast A. (2008). Health effects of quercetin: From antioxidant to nutraceutical. Eur. J. Pharmacol..

[34] Zhao Y., Tang Y., Sang S. (2021). Dietary Quercetin Reduces Plasma and Tissue Methylglyoxal and Advanced Glycation End Products in Healthy Mice Treated with Methylglyoxal. J. Nutr..

[35] Li X., Zheng T., Sang S., Lv L. (2014). Quercetin Inhibits Advanced Glycation End Product Formation by Trapping Methylglyoxal and Glyoxal. J. Agric. Food Chem..

[36] Yeh W.-J., Hsia S.-M., Lee W.-H., Wu C.-H. (2017). Polyphenols with antiglycation activity and mechanisms of action: A review of recent findings. J. Food Drug Anal..

[37] Núñez S., Moliner C., Valero M.S., Mustafa A.M., Maggi F., Gómez-Rincón C., López V. (2023). Antidiabetic and anti-obesity properties of a polyphenol-rich flower extract from *Tagetes erecta* L. and its effects on Caenorhabditis elegans fat storages. J. Physiol. Biochem..

[38] Modanwal S., Maurya A.K., Mishra S.K., Mishra N. (2022). Development of QSAR model using machine learning and molecular docking study of polyphenol derivatives against obesity as pancreatic lipase inhibitor. J. Biomol. Struct. Dyn..

[39] Bialecka-Florjanczyk E., Fabiszewska A.U., Krzyczkowska J., Kurylowicz A. (2018). Synthetic and Natural Lipase Inhibitors. Mini Rev. Med. Chem..

