# Supplementary material for: Antioxidant, Antidiabetic, and Anti-Obesity Properties of Apple Pulp Extracts (Malus domestica Bork): A Comparative Study of 15 Local and Commercial Cultivars from Spain
Source: Biology (Basel). 2023 Jun 21;12(7):891. doi: 10.3390/biology12070891 (PMC10376420; doi:10.3390/biology12070891)
Supplement: Supplementary file 1 [file biology-12-00891-s001.zip › biology-2425240-supplementary.pdf]

Table S1. Folin-Ciocalteu data of the 15 apple pulp extracts expressed as the mean of milligrams of gallic acid per gram of dry weight extract of each repetition. Note: Outlier points are marked. * $p < 0.05$.

Sample	Values (µg gallic acid/gram of extract)
Arraiza	8.98
	10.42
	7.53
	8.23
De Mine	7.02
	6.69
	7.59
	6.05
Gordoncha	6.98
	5.54
	5.64
	6.15
Ziordia	17.55
	14.99
	16.67
	14.65
M. Tomate	10.43
	8.10
	9.20
	8.62
Pomera del pais	3.43
	4.53
	5.42
	4.01
MA-190	7.57
	7.26
	6.61
	6.94
Manzana helada	10.54
	8.73
	8.91
	10.26
Pomera de Pomes agrias	18.02
	17.45
	18.49
	17.47
Doncella de San Martin	12.09
	13.03
	15.83
	13.02
Esperiega de Ademuz	6.37
	5.48
	4.50
	5.67
Amarilla de Octubre	32.82
	21.98

	26.90
	42.50*
Royal Gala	-
	1.92*
	5.34
	5.33
V. Doncella	6.01
	5.51
	6.75
	5.97
Pinova	4.59
	4.37
	5.07
	4.23
